# Supplementary material for: Pyruvate Dehydrogenase Kinase 1 inhibition mediated oxidative phosphorylation enhancement in cartilage promotes osteoarthritis progression
Source: BMC Musculoskelet Disord. 2023 Jul 20;24:597. doi: 10.1186/s12891-023-06585-6 (PMC10357736; doi:10.1186/s12891-023-06585-6)
Supplement: Supplementary file 1 — Additional file 1. [file 12891_2023_6585_MOESM1_ESM.zip › MF.Gsea.1653627316242/GOMF_HISTONE_BINDING.html]

Details for gene set GOMF\_HISTONE\_BINDING[GSEA]

|  || Dataset | 55457\_collapsed\_to\_symbols.cls.cls #OA\_versus\_normal.cls.cls #OA\_versus\_normal\_repos |
| Phenotype | cls.cls#OA\_versus\_normal\_repos |
| Upregulated in class | normal |
| GeneSet | GOMF\_HISTONE\_BINDING |
| Enrichment Score (ES) | -0.35668626 |
| Normalized Enrichment Score (NES) | -1.6786005 |
| Nominal p-value | 0.0 |
| FDR q-value | 0.11461958 |
| FWER p-Value | 0.937 |
Table: GSEA Results Summary

  

Fig 1: Enrichment plot: GOMF\_HISTONE\_BINDING      
 Profile of the Running ES Score & Positions of GeneSet Members on the Rank Ordered List

  

| SYMBOL | TITLE | RANK IN GENE LIST | RANK METRIC SCORE | RUNNING ES | CORE ENRICHMENT || 1 | ASF1A | anti-silencing function 1A histone chaperone [Source:HGNC Symbol;Acc:HGNC:20995] | 88 | 0.928 | 0.0067 | No |
| 2 | NAP1L3 | nucleosome assembly protein 1 like 3 [Source:HGNC Symbol;Acc:HGNC:7639] | 229 | 0.800 | 0.0074 | No |
| 3 | ANP32A | acidic nuclear phosphoprotein 32 family member A [Source:HGNC Symbol;Acc:HGNC:13233] | 400 | 0.707 | 0.0044 | No |
| 4 | VPS72 | vacuolar protein sorting 72 homolog [Source:HGNC Symbol;Acc:HGNC:11644] | 533 | 0.647 | 0.0035 | No |
| 5 | KAT8 | lysine acetyltransferase 8 [Source:HGNC Symbol;Acc:HGNC:17933] | 595 | 0.625 | 0.0079 | No |
| 6 | ING4 | inhibitor of growth family member 4 [Source:HGNC Symbol;Acc:HGNC:19423] | 680 | 0.598 | 0.0100 | No |
| 7 | BRD7 | bromodomain containing 7 [Source:HGNC Symbol;Acc:HGNC:14310] | 726 | 0.588 | 0.0151 | No |
| 8 | MLLT3 | MLLT3 super elongation complex subunit [Source:HGNC Symbol;Acc:HGNC:7136] | 764 | 0.577 | 0.0206 | No |
| 9 | PHF10 | PHD finger protein 10 [Source:HGNC Symbol;Acc:HGNC:18250] | 842 | 0.557 | 0.0228 | No |
| 10 | DPF2 | double PHD fingers 2 [Source:HGNC Symbol;Acc:HGNC:9964] | 854 | 0.554 | 0.0300 | No |
| 11 | NPM3 | nucleophosmin/nucleoplasmin 3 [Source:HGNC Symbol;Acc:HGNC:7931] | 868 | 0.551 | 0.0371 | No |
| 12 | MCM2 | minichromosome maintenance complex component 2 [Source:HGNC Symbol;Acc:HGNC:6944] | 888 | 0.546 | 0.0436 | No |
| 13 | CKS1B | CDC28 protein kinase regulatory subunit 1B [Source:HGNC Symbol;Acc:HGNC:19083] | 927 | 0.533 | 0.0484 | No |
| 14 | NAP1L2 | nucleosome assembly protein 1 like 2 [Source:HGNC Symbol;Acc:HGNC:7638] | 935 | 0.531 | 0.0556 | No |
| 15 | HPF1 | histone PARylation factor 1 [Source:HGNC Symbol;Acc:HGNC:26051] | 965 | 0.525 | 0.0611 | No |
| 16 | WDR5B | WD repeat domain 5B [Source:HGNC Symbol;Acc:HGNC:17826] | 1038 | 0.510 | 0.0629 | No |
| 17 | TBL1XR1 | TBL1X receptor 1 [Source:HGNC Symbol;Acc:HGNC:29529] | 1070 | 0.501 | 0.0678 | No |
| 18 | KAT7 | lysine acetyltransferase 7 [Source:HGNC Symbol;Acc:HGNC:17016] | 1125 | 0.491 | 0.0707 | No |
| 19 | RBBP4 | "RB binding protein 4, chromatin remodeling factor [Source:HGNC Symbol;Acc:HGNC:9887]" | 1181 | 0.480 | 0.0734 | No |
| 20 | CBX1 | chromobox 1 [Source:HGNC Symbol;Acc:HGNC:1551] | 1331 | 0.453 | 0.0684 | No |
| 21 | TSPYL5 | TSPY like 5 [Source:HGNC Symbol;Acc:HGNC:29367] | 1338 | 0.452 | 0.0745 | No |
| 22 | CBX4 | chromobox 4 [Source:HGNC Symbol;Acc:HGNC:1554] | 1397 | 0.443 | 0.0764 | No |
| 23 | BCAS3 | BCAS3 microtubule associated cell migration factor [Source:HGNC Symbol;Acc:HGNC:14347] | 1471 | 0.433 | 0.0770 | No |
| 24 | RRP8 | ribosomal RNA processing 8 [Source:HGNC Symbol;Acc:HGNC:29030] | 1673 | 0.402 | 0.0671 | No |
| 25 | TSPYL4 | TSPY like 4 [Source:HGNC Symbol;Acc:HGNC:21559] | 1713 | 0.396 | 0.0699 | No |
| 26 | DNAJC9 | DnaJ heat shock protein family (Hsp40) member C9 [Source:HGNC Symbol;Acc:HGNC:19123] | 1715 | 0.396 | 0.0756 | No |
| 27 | SNCA | synuclein alpha [Source:HGNC Symbol;Acc:HGNC:11138] | 1800 | 0.384 | 0.0746 | No |
| 28 | BRDT | bromodomain testis associated [Source:HGNC Symbol;Acc:HGNC:1105] | 1812 | 0.382 | 0.0794 | No |
| 29 | HAT1 | histone acetyltransferase 1 [Source:HGNC Symbol;Acc:HGNC:4821] | 2118 | 0.348 | 0.0605 | No |
| 30 | NCAPD2 | non-SMC condensin I complex subunit D2 [Source:HGNC Symbol;Acc:HGNC:24305] | 2271 | 0.329 | 0.0533 | No |
| 31 | PKN1 | protein kinase N1 [Source:HGNC Symbol;Acc:HGNC:9405] | 2540 | 0.298 | 0.0366 | No |
| 32 | KDM8 | lysine demethylase 8 [Source:HGNC Symbol;Acc:HGNC:25840] | 2594 | 0.291 | 0.0367 | No |
| 33 | PIH1D1 | PIH1 domain containing 1 [Source:HGNC Symbol;Acc:HGNC:26075] | 2667 | 0.283 | 0.0352 | No |
| 34 | RBBP7 | "RB binding protein 7, chromatin remodeling factor [Source:HGNC Symbol;Acc:HGNC:9890]" | 2777 | 0.272 | 0.0306 | No |
| 35 | KAT6B | lysine acetyltransferase 6B [Source:HGNC Symbol;Acc:HGNC:17582] | 2827 | 0.267 | 0.0307 | No |
| 36 | YEATS4 | YEATS domain containing 4 [Source:HGNC Symbol;Acc:HGNC:24859] | 2933 | 0.257 | 0.0262 | No |
| 37 | TAF7 | TATA-box binding protein associated factor 7 [Source:HGNC Symbol;Acc:HGNC:11541] | 3035 | 0.246 | 0.0219 | No |
| 38 | ASF1B | anti-silencing function 1B histone chaperone [Source:HGNC Symbol;Acc:HGNC:20996] | 3221 | 0.228 | 0.0107 | No |
| 39 | PRMT7 | protein arginine methyltransferase 7 [Source:HGNC Symbol;Acc:HGNC:25557] | 3250 | 0.226 | 0.0118 | No |
| 40 | SMARCA2 | "SWI/SNF related, matrix associated, actin dependent regulator of chromatin, subfamily a, member 2 [Source:HGNC Symbol;Acc:HGNC:11098]" | 3281 | 0.222 | 0.0127 | No |
| 41 | ANP32C | acidic nuclear phosphoprotein 32 family member C [Source:HGNC Symbol;Acc:HGNC:16675] | 3450 | 0.207 | 0.0025 | No |
| 42 | CD1D | CD1d molecule [Source:HGNC Symbol;Acc:HGNC:1637] | 3516 | 0.201 | 0.0003 | No |
| 43 | ATAD2 | ATPase family AAA domain containing 2 [Source:HGNC Symbol;Acc:HGNC:30123] | 3597 | 0.194 | -0.0031 | No |
| 44 | TNKS | tankyrase [Source:HGNC Symbol;Acc:HGNC:11941] | 3622 | 0.192 | -0.0022 | No |
| 45 | USP16 | ubiquitin specific peptidase 16 [Source:HGNC Symbol;Acc:HGNC:12614] | 3831 | 0.173 | -0.0160 | No |
| 46 | WDTC1 | WD and tetratricopeptide repeats 1 [Source:HGNC Symbol;Acc:HGNC:29175] | 4121 | 0.144 | -0.0366 | No |
| 47 | JAK2 | Janus kinase 2 [Source:HGNC Symbol;Acc:HGNC:6192] | 4183 | 0.140 | -0.0394 | No |
| 48 | STAT1 | signal transducer and activator of transcription 1 [Source:HGNC Symbol;Acc:HGNC:11362] | 4495 | 0.113 | -0.0622 | No |
| 49 | MORC4 | MORC family CW-type zinc finger 4 [Source:HGNC Symbol;Acc:HGNC:23485] | 4525 | 0.111 | -0.0628 | No |
| 50 | NCAPD3 | non-SMC condensin II complex subunit D3 [Source:HGNC Symbol;Acc:HGNC:28952] | 4711 | 0.095 | -0.0760 | No |
| 51 | TSPYL1 | TSPY like 1 [Source:HGNC Symbol;Acc:HGNC:12382] | 4829 | 0.085 | -0.0839 | No |
| 52 | RNF8 | ring finger protein 8 [Source:HGNC Symbol;Acc:HGNC:10071] | 4938 | 0.077 | -0.0913 | No |
| 53 | TP53BP1 | tumor protein p53 binding protein 1 [Source:HGNC Symbol;Acc:HGNC:11999] | 4946 | 0.076 | -0.0907 | No |
| 54 | CBX5 | chromobox 5 [Source:HGNC Symbol;Acc:HGNC:1555] | 4963 | 0.075 | -0.0909 | No |
| 55 | CDYL | chromodomain Y like [Source:HGNC Symbol;Acc:HGNC:1811] | 5016 | 0.072 | -0.0939 | No |
| 56 | SAP30L | SAP30 like [Source:HGNC Symbol;Acc:HGNC:25663] | 5124 | 0.062 | -0.1014 | No |
| 57 | ING1 | inhibitor of growth family member 1 [Source:HGNC Symbol;Acc:HGNC:6062] | 5146 | 0.060 | -0.1022 | No |
| 58 | TSPY1 | testis specific protein Y-linked 1 [Source:HGNC Symbol;Acc:HGNC:12381] | 5214 | 0.055 | -0.1067 | No |
| 59 | PHF2 | PHD finger protein 2 [Source:HGNC Symbol;Acc:HGNC:8920] | 5332 | 0.045 | -0.1152 | No |
| 60 | KDM5B | lysine demethylase 5B [Source:HGNC Symbol;Acc:HGNC:18039] | 5484 | 0.033 | -0.1266 | No |
| 61 | MSL3 | MSL complex subunit 3 [Source:HGNC Symbol;Acc:HGNC:7370] | 5596 | 0.024 | -0.1350 | No |
| 62 | SPIN2A | spindlin family member 2A [Source:HGNC Symbol;Acc:HGNC:20694] | 5689 | 0.017 | -0.1420 | No |
| 63 | NCAPG2 | non-SMC condensin II complex subunit G2 [Source:HGNC Symbol;Acc:HGNC:21904] | 5700 | 0.016 | -0.1425 | No |
| 64 | KAT6A | lysine acetyltransferase 6A [Source:HGNC Symbol;Acc:HGNC:13013] | 5778 | 0.010 | -0.1484 | No |
| 65 | DPF3 | double PHD fingers 3 [Source:HGNC Symbol;Acc:HGNC:17427] | 5907 | 0.002 | -0.1585 | No |
| 66 | MTF2 | metal response element binding transcription factor 2 [Source:HGNC Symbol;Acc:HGNC:29535] | 5996 | -0.004 | -0.1653 | No |
| 67 | ING2 | inhibitor of growth family member 2 [Source:HGNC Symbol;Acc:HGNC:6063] | 6150 | -0.016 | -0.1771 | No |
| 68 | NAP1L4 | nucleosome assembly protein 1 like 4 [Source:HGNC Symbol;Acc:HGNC:7640] | 6289 | -0.027 | -0.1876 | No |
| 69 | SCMH1 | Scm polycomb group protein homolog 1 [Source:HGNC Symbol;Acc:HGNC:19003] | 6363 | -0.032 | -0.1929 | No |
| 70 | KAT5 | lysine acetyltransferase 5 [Source:HGNC Symbol;Acc:HGNC:5275] | 6379 | -0.034 | -0.1935 | No |
| 71 | THAP7 | THAP domain containing 7 [Source:HGNC Symbol;Acc:HGNC:23190] | 6412 | -0.036 | -0.1955 | No |
| 72 | PRKCB | protein kinase C beta [Source:HGNC Symbol;Acc:HGNC:9395] | 6453 | -0.039 | -0.1981 | No |
| 73 | ING3 | inhibitor of growth family member 3 [Source:HGNC Symbol;Acc:HGNC:14587] | 6518 | -0.043 | -0.2025 | No |
| 74 | HINFP | histone H4 transcription factor [Source:HGNC Symbol;Acc:HGNC:17850] | 6776 | -0.061 | -0.2218 | No |
| 75 | RAG1 | recombination activating 1 [Source:HGNC Symbol;Acc:HGNC:9831] | 6806 | -0.063 | -0.2232 | No |
| 76 | L3MBTL1 | L3MBTL histone methyl-lysine binding protein 1 [Source:HGNC Symbol;Acc:HGNC:15905] | 6807 | -0.063 | -0.2222 | No |
| 77 | ATAD2B | ATPase family AAA domain containing 2B [Source:HGNC Symbol;Acc:HGNC:29230] | 6896 | -0.072 | -0.2281 | No |
| 78 | SGF29 | SAGA complex associated factor 29 [Source:HGNC Symbol;Acc:HGNC:25156] | 7363 | -0.107 | -0.2632 | No |
| 79 | RSF1 | remodeling and spacing factor 1 [Source:HGNC Symbol;Acc:HGNC:18118] | 7393 | -0.110 | -0.2639 | No |
| 80 | MPHOSPH8 | M-phase phosphoprotein 8 [Source:HGNC Symbol;Acc:HGNC:29810] | 7413 | -0.112 | -0.2637 | No |
| 81 | HJURP | Holliday junction recognition protein [Source:HGNC Symbol;Acc:HGNC:25444] | 7419 | -0.112 | -0.2625 | No |
| 82 | PHF1 | PHD finger protein 1 [Source:HGNC Symbol;Acc:HGNC:8919] | 7429 | -0.113 | -0.2615 | No |
| 83 | CHAF1B | chromatin assembly factor 1 subunit B [Source:HGNC Symbol;Acc:HGNC:1911] | 7461 | -0.115 | -0.2623 | No |
| 84 | RAG2 | recombination activating 2 [Source:HGNC Symbol;Acc:HGNC:9832] | 7462 | -0.115 | -0.2606 | No |
| 85 | LEF1 | lymphoid enhancer binding factor 1 [Source:HGNC Symbol;Acc:HGNC:6551] | 7467 | -0.116 | -0.2592 | No |
| 86 | ZZEF1 | zinc finger ZZ-type and EF-hand domain containing 1 [Source:HGNC Symbol;Acc:HGNC:29027] | 7490 | -0.118 | -0.2592 | No |
| 87 | ATRX | ATRX chromatin remodeler [Source:HGNC Symbol;Acc:HGNC:886] | 7551 | -0.123 | -0.2621 | No |
| 88 | FMR1 | FMRP translational regulator 1 [Source:HGNC Symbol;Acc:HGNC:3775] | 7944 | -0.151 | -0.2907 | No |
| 89 | DEK | DEK proto-oncogene [Source:HGNC Symbol;Acc:HGNC:2768] | 7992 | -0.154 | -0.2922 | No |
| 90 | SMARCC1 | "SWI/SNF related, matrix associated, actin dependent regulator of chromatin subfamily c member 1 [Source:HGNC Symbol;Acc:HGNC:11104]" | 8052 | -0.159 | -0.2945 | No |
| 91 | WRAP53 | WD repeat containing antisense to TP53 [Source:HGNC Symbol;Acc:HGNC:25522] | 8055 | -0.160 | -0.2923 | No |
| 92 | MCM3AP | minichromosome maintenance complex component 3 associated protein [Source:HGNC Symbol;Acc:HGNC:6946] | 8077 | -0.161 | -0.2916 | No |
| 93 | CDY1 | chromodomain Y-linked 1 [Source:HGNC Symbol;Acc:HGNC:1809] | 8096 | -0.163 | -0.2906 | No |
| 94 | CARM1 | coactivator associated arginine methyltransferase 1 [Source:HGNC Symbol;Acc:HGNC:23393] | 8131 | -0.165 | -0.2909 | No |
| 95 | BRD3 | bromodomain containing 3 [Source:HGNC Symbol;Acc:HGNC:1104] | 8369 | -0.183 | -0.3068 | No |
| 96 | SMARCC2 | "SWI/SNF related, matrix associated, actin dependent regulator of chromatin subfamily c member 2 [Source:HGNC Symbol;Acc:HGNC:11105]" | 8480 | -0.192 | -0.3126 | No |
| 97 | BRD9 | bromodomain containing 9 [Source:HGNC Symbol;Acc:HGNC:25818] | 8483 | -0.193 | -0.3100 | No |
| 98 | USP15 | ubiquitin specific peptidase 15 [Source:HGNC Symbol;Acc:HGNC:12613] | 8653 | -0.205 | -0.3203 | No |
| 99 | SSRP1 | structure specific recognition protein 1 [Source:HGNC Symbol;Acc:HGNC:11327] | 8692 | -0.208 | -0.3202 | No |
| 100 | PWP1 | "PWP1 homolog, endonuclein [Source:HGNC Symbol;Acc:HGNC:17015]" | 8724 | -0.211 | -0.3195 | No |
| 101 | SUPT6H | "SPT6 homolog, histone chaperone and transcription elongation factor [Source:HGNC Symbol;Acc:HGNC:11470]" | 8752 | -0.213 | -0.3186 | No |
| 102 | KMT2A | lysine methyltransferase 2A [Source:HGNC Symbol;Acc:HGNC:7132] | 8807 | -0.217 | -0.3196 | No |
| 103 | MSH6 | mutS homolog 6 [Source:HGNC Symbol;Acc:HGNC:7329] | 8846 | -0.221 | -0.3194 | No |
| 104 | ZCWPW1 | zinc finger CW-type and PWWP domain containing 1 [Source:HGNC Symbol;Acc:HGNC:23486] | 8865 | -0.222 | -0.3175 | No |
| 105 | DAXX | death domain associated protein [Source:HGNC Symbol;Acc:HGNC:2681] | 8926 | -0.226 | -0.3189 | No |
| 106 | SIRT1 | sirtuin 1 [Source:HGNC Symbol;Acc:HGNC:14929] | 8947 | -0.227 | -0.3172 | No |
| 107 | ZMYND11 | zinc finger MYND-type containing 11 [Source:HGNC Symbol;Acc:HGNC:16966] | 8952 | -0.228 | -0.3141 | No |
| 108 | H2AX | H2A.X variant histone [Source:HGNC Symbol;Acc:HGNC:4739] | 8979 | -0.230 | -0.3128 | No |
| 109 | ZZZ3 | zinc finger ZZ-type containing 3 [Source:HGNC Symbol;Acc:HGNC:24523] | 8991 | -0.231 | -0.3103 | No |
| 110 | TDRD3 | tudor domain containing 3 [Source:HGNC Symbol;Acc:HGNC:20612] | 9080 | -0.238 | -0.3137 | No |
| 111 | TSPYL2 | TSPY like 2 [Source:HGNC Symbol;Acc:HGNC:24358] | 9491 | -0.273 | -0.3420 | No |
| 112 | TSPY2 | testis specific protein Y-linked 2 [Source:HGNC Symbol;Acc:HGNC:23924] | 9518 | -0.276 | -0.3400 | No |
| 113 | USP3 | ubiquitin specific peptidase 3 [Source:HGNC Symbol;Acc:HGNC:12626] | 9567 | -0.280 | -0.3396 | No |
| 114 | ANP32E | acidic nuclear phosphoprotein 32 family member E [Source:HGNC Symbol;Acc:HGNC:16673] | 9568 | -0.280 | -0.3355 | No |
| 115 | PYGO1 | pygopus family PHD finger 1 [Source:HGNC Symbol;Acc:HGNC:30256] | 9628 | -0.286 | -0.3360 | No |
| 116 | RESF1 | retroelement silencing factor 1 [Source:HGNC Symbol;Acc:HGNC:25559] | 9814 | -0.302 | -0.3461 | No |
| 117 | TRIM24 | tripartite motif containing 24 [Source:HGNC Symbol;Acc:HGNC:11812] | 9927 | -0.314 | -0.3503 | No |
| 118 | SMARCA4 | "SWI/SNF related, matrix associated, actin dependent regulator of chromatin, subfamily a, member 4 [Source:HGNC Symbol;Acc:HGNC:11100]" | 10009 | -0.322 | -0.3520 | Yes |
| 119 | MORC3 | MORC family CW-type zinc finger 3 [Source:HGNC Symbol;Acc:HGNC:23572] | 10037 | -0.325 | -0.3493 | Yes |
| 120 | CBX2 | chromobox 2 [Source:HGNC Symbol;Acc:HGNC:1552] | 10065 | -0.327 | -0.3466 | Yes |
| 121 | DNAJC2 | DnaJ heat shock protein family (Hsp40) member C2 [Source:HGNC Symbol;Acc:HGNC:13192] | 10081 | -0.329 | -0.3430 | Yes |
| 122 | SFMBT1 | Scm like with four mbt domains 1 [Source:HGNC Symbol;Acc:HGNC:20255] | 10094 | -0.330 | -0.3391 | Yes |
| 123 | SUZ12 | SUZ12 polycomb repressive complex 2 subunit [Source:HGNC Symbol;Acc:HGNC:17101] | 10104 | -0.331 | -0.3350 | Yes |
| 124 | BAZ1B | bromodomain adjacent to zinc finger domain 1B [Source:HGNC Symbol;Acc:HGNC:961] | 10112 | -0.332 | -0.3306 | Yes |
| 125 | ANP32B | acidic nuclear phosphoprotein 32 family member B [Source:HGNC Symbol;Acc:HGNC:16677] | 10327 | -0.353 | -0.3423 | Yes |
| 126 | CHD2 | chromodomain helicase DNA binding protein 2 [Source:HGNC Symbol;Acc:HGNC:1917] | 10467 | -0.370 | -0.3478 | Yes |
| 127 | PHC1 | polyhomeotic homolog 1 [Source:HGNC Symbol;Acc:HGNC:3182] | 10486 | -0.372 | -0.3438 | Yes |
| 128 | MLLT10 | MLLT10 histone lysine methyltransferase DOT1L cofactor [Source:HGNC Symbol;Acc:HGNC:16063] | 10645 | -0.387 | -0.3505 | Yes |
| 129 | IPO7 | importin 7 [Source:HGNC Symbol;Acc:HGNC:9852] | 10662 | -0.389 | -0.3461 | Yes |
| 130 | PSME4 | proteasome activator subunit 4 [Source:HGNC Symbol;Acc:HGNC:20635] | 10694 | -0.392 | -0.3428 | Yes |
| 131 | CKS2 | CDC28 protein kinase regulatory subunit 2 [Source:HGNC Symbol;Acc:HGNC:2000] | 10695 | -0.392 | -0.3370 | Yes |
| 132 | PHIP | pleckstrin homology domain interacting protein [Source:HGNC Symbol;Acc:HGNC:15673] | 10715 | -0.395 | -0.3327 | Yes |
| 133 | GLYR1 | glyoxylate reductase 1 homolog [Source:HGNC Symbol;Acc:HGNC:24434] | 10807 | -0.406 | -0.3339 | Yes |
| 134 | CTSL | cathepsin L [Source:HGNC Symbol;Acc:HGNC:2537] | 10817 | -0.407 | -0.3287 | Yes |
| 135 | CXXC1 | CXXC finger protein 1 [Source:HGNC Symbol;Acc:HGNC:24343] | 10853 | -0.411 | -0.3254 | Yes |
| 136 | TTLL12 | tubulin tyrosine ligase like 12 [Source:HGNC Symbol;Acc:HGNC:28974] | 10861 | -0.411 | -0.3199 | Yes |
| 137 | SBNO1 | strawberry notch homolog 1 [Source:HGNC Symbol;Acc:HGNC:22973] | 10924 | -0.421 | -0.3186 | Yes |
| 138 | KDM4A | lysine demethylase 4A [Source:HGNC Symbol;Acc:HGNC:22978] | 10936 | -0.423 | -0.3133 | Yes |
| 139 | KDM5A | lysine demethylase 5A [Source:HGNC Symbol;Acc:HGNC:9886] | 11097 | -0.443 | -0.3194 | Yes |
| 140 | CBX3 | chromobox 3 [Source:HGNC Symbol;Acc:HGNC:1553] | 11113 | -0.445 | -0.3140 | Yes |
| 141 | TBL1X | transducin beta like 1 X-linked [Source:HGNC Symbol;Acc:HGNC:11585] | 11263 | -0.466 | -0.3189 | Yes |
| 142 | BRD1 | bromodomain containing 1 [Source:HGNC Symbol;Acc:HGNC:1102] | 11549 | -0.507 | -0.3339 | Yes |
| 143 | SCML2 | Scm polycomb group protein like 2 [Source:HGNC Symbol;Acc:HGNC:10581] | 11567 | -0.510 | -0.3278 | Yes |
| 144 | KMT2D | lysine methyltransferase 2D [Source:HGNC Symbol;Acc:HGNC:7133] | 11590 | -0.514 | -0.3220 | Yes |
| 145 | PHF8 | PHD finger protein 8 [Source:HGNC Symbol;Acc:HGNC:20672] | 11592 | -0.514 | -0.3145 | Yes |
| 146 | PTMS | parathymosin [Source:HGNC Symbol;Acc:HGNC:9629] | 11632 | -0.522 | -0.3099 | Yes |
| 147 | NAP1L1 | nucleosome assembly protein 1 like 1 [Source:HGNC Symbol;Acc:HGNC:7637] | 11685 | -0.530 | -0.3062 | Yes |
| 148 | GRWD1 | glutamate rich WD repeat containing 1 [Source:HGNC Symbol;Acc:HGNC:21270] | 11694 | -0.531 | -0.2991 | Yes |
| 149 | PHC2 | polyhomeotic homolog 2 [Source:HGNC Symbol;Acc:HGNC:3183] | 11701 | -0.533 | -0.2917 | Yes |
| 150 | CHD5 | chromodomain helicase DNA binding protein 5 [Source:HGNC Symbol;Acc:HGNC:16816] | 11776 | -0.545 | -0.2896 | Yes |
| 151 | RBBP5 | "RB binding protein 5, histone lysine methyltransferase complex subunit [Source:HGNC Symbol;Acc:HGNC:9888]" | 11816 | -0.553 | -0.2845 | Yes |
| 152 | CHD8 | chromodomain helicase DNA binding protein 8 [Source:HGNC Symbol;Acc:HGNC:20153] | 11825 | -0.554 | -0.2770 | Yes |
| 153 | SMARCA5 | "SWI/SNF related, matrix associated, actin dependent regulator of chromatin, subfamily a, member 5 [Source:HGNC Symbol;Acc:HGNC:11101]" | 11838 | -0.557 | -0.2698 | Yes |
| 154 | SBNO2 | strawberry notch homolog 2 [Source:HGNC Symbol;Acc:HGNC:29158] | 11850 | -0.560 | -0.2625 | Yes |
| 155 | MLLT1 | MLLT1 super elongation complex subunit [Source:HGNC Symbol;Acc:HGNC:7134] | 11857 | -0.561 | -0.2547 | Yes |
| 156 | BPTF | bromodomain PHD finger transcription factor [Source:HGNC Symbol;Acc:HGNC:3581] | 11870 | -0.565 | -0.2474 | Yes |
| 157 | PHC3 | polyhomeotic homolog 3 [Source:HGNC Symbol;Acc:HGNC:15682] | 11881 | -0.568 | -0.2399 | Yes |
| 158 | SCML1 | Scm polycomb group protein like 1 [Source:HGNC Symbol;Acc:HGNC:10580] | 11890 | -0.570 | -0.2321 | Yes |
| 159 | UIMC1 | ubiquitin interaction motif containing 1 [Source:HGNC Symbol;Acc:HGNC:30298] | 11919 | -0.576 | -0.2259 | Yes |
| 160 | NASP | nuclear autoantigenic sperm protein [Source:HGNC Symbol;Acc:HGNC:7644] | 11970 | -0.587 | -0.2212 | Yes |
| 161 | SET | SET nuclear proto-oncogene [Source:HGNC Symbol;Acc:HGNC:10760] | 12000 | -0.593 | -0.2148 | Yes |
| 162 | JARID2 | jumonji and AT-rich interaction domain containing 2 [Source:HGNC Symbol;Acc:HGNC:6196] | 12030 | -0.600 | -0.2083 | Yes |
| 163 | HIRA | histone cell cycle regulator [Source:HGNC Symbol;Acc:HGNC:4916] | 12143 | -0.630 | -0.2079 | Yes |
| 164 | SART3 | "spliceosome associated factor 3, U4/U6 recycling protein [Source:HGNC Symbol;Acc:HGNC:16860]" | 12161 | -0.633 | -0.1999 | Yes |
| 165 | YEATS2 | YEATS domain containing 2 [Source:HGNC Symbol;Acc:HGNC:25489] | 12179 | -0.639 | -0.1919 | Yes |
| 166 | MBTD1 | mbt domain containing 1 [Source:HGNC Symbol;Acc:HGNC:19866] | 12189 | -0.641 | -0.1832 | Yes |
| 167 | ZMYND8 | zinc finger MYND-type containing 8 [Source:HGNC Symbol;Acc:HGNC:9397] | 12214 | -0.649 | -0.1756 | Yes |
| 168 | NOC2L | NOC2 like nucleolar associated transcriptional repressor [Source:HGNC Symbol;Acc:HGNC:24517] | 12258 | -0.663 | -0.1692 | Yes |
| 169 | KDM7A | lysine demethylase 7A [Source:HGNC Symbol;Acc:HGNC:22224] | 12390 | -0.704 | -0.1692 | Yes |
| 170 | NPM1 | nucleophosmin 1 [Source:HGNC Symbol;Acc:HGNC:7910] | 12530 | -0.762 | -0.1690 | Yes |
| 171 | SRCAP | Snf2 related CREBBP activator protein [Source:HGNC Symbol;Acc:HGNC:16974] | 12542 | -0.769 | -0.1586 | Yes |
| 172 | SPIN1 | spindlin 1 [Source:HGNC Symbol;Acc:HGNC:11243] | 12559 | -0.777 | -0.1484 | Yes |
| 173 | IPO9 | importin 9 [Source:HGNC Symbol;Acc:HGNC:19425] | 12575 | -0.783 | -0.1381 | Yes |
| 174 | CBX8 | chromobox 8 [Source:HGNC Symbol;Acc:HGNC:15962] | 12586 | -0.787 | -0.1274 | Yes |
| 175 | BRD4 | bromodomain containing 4 [Source:HGNC Symbol;Acc:HGNC:13575] | 12606 | -0.800 | -0.1172 | Yes |
| 176 | AIRE | autoimmune regulator [Source:HGNC Symbol;Acc:HGNC:360] | 12610 | -0.804 | -0.1056 | Yes |
| 177 | DPF1 | double PHD fingers 1 [Source:HGNC Symbol;Acc:HGNC:20225] | 12671 | -0.844 | -0.0979 | Yes |
| 178 | RCC1 | regulator of chromosome condensation 1 [Source:HGNC Symbol;Acc:HGNC:1913] | 12699 | -0.864 | -0.0874 | Yes |
| 179 | BRD2 | bromodomain containing 2 [Source:HGNC Symbol;Acc:HGNC:1103] | 12709 | -0.871 | -0.0754 | Yes |
| 180 | PTMA | prothymosin alpha [Source:HGNC Symbol;Acc:HGNC:9623] | 12742 | -0.906 | -0.0646 | Yes |
| 181 | CHD1 | chromodomain helicase DNA binding protein 1 [Source:HGNC Symbol;Acc:HGNC:1915] | 12745 | -0.908 | -0.0514 | Yes |
| 182 | AASS | aminoadipate-semialdehyde synthase [Source:HGNC Symbol;Acc:HGNC:17366] | 12799 | -0.968 | -0.0414 | Yes |
| 183 | APBB1 | amyloid beta precursor protein binding family B member 1 [Source:HGNC Symbol;Acc:HGNC:581] | 12811 | -0.992 | -0.0277 | Yes |
| 184 | BAZ2A | bromodomain adjacent to zinc finger domain 2A [Source:HGNC Symbol;Acc:HGNC:962] | 12879 | -1.166 | -0.0159 | Yes |
| 185 | TAF1 | TATA-box binding protein associated factor 1 [Source:HGNC Symbol;Acc:HGNC:11535] | 12881 | -1.192 | 0.0015 | Yes |
Table: GSEA details [plain text format]

  

Fig 2: GOMF\_HISTONE\_BINDING      
 Blue-Pink O' Gram in the Space of the Analyzed GeneSet

  

Fig 3: GOMF\_HISTONE\_BINDING: Random ES distribution      
 Gene set null distribution of ES for **GOMF\_HISTONE\_BINDING**
